# Breast tuberculosis: a case series

**DOI:** 10.1186/s13256-020-02646-9

**Published:** 2021-02-20

**Authors:** M. Ghalleb, S. Seghaier, O. Adouni, H. Bouaziz, A. Bouida, J. Ben Hassouna, R. Chargui, K. Rahal

**Affiliations:** 1 Surgical Oncology Department, Institute Salah Azaiez of Oncology, Tunis, Tunisia; 2 Immuno-Histo-Cytology Department, Institute Salah Azaiez of Oncology, Tunis, Tunisia; 3 Faculté de Médecine de Tunis, Institut Salah Azaiz, Université Tunis el Manar, Boulevard Avril 1938, 1006 Tunis, Tunisia

**Keywords:** Tuberculosis, Breast disease, Granulomatosis

## Abstract

**Background and aim:**

Breast tuberculosis is a rare disease, even in endemic areas. The diagnosis can be challenging, as it can mimic breast cancer. We aim to report our experience and discuss diagnoses and management modalities.

**Results:**

We encountered twelve cases of breast tuberculosis in our institution from 2004 to 2019. The average age of our Caucasian North African patients was 42 years old (22–63). The classic presentation was a breast lump found in half of the cases. On physical examination, we suspected breast carcinoma in seven patients. The average size of the tumors was 39 mm (15–80 mm). Nine patients had a mammogram. In five cases, there was a suspicious breast mass mimicking a malignant tumor with an average size of 33 mm (25–60 mm). A ultrasonography was performed in 6 cases and revealed a suspicious ill-circumscribed nodule in four patients with an average size of 37.5 mm (10–60 mm). Five patients had a lumpectomy, and seven women underwent drainage of the abscess and the biopsy of its hull. The association of epithelioid cell granulomas and caseous necrosis was mandatory for the histological diagnosis of tuberculosis. All of them had an antitubercular therapy. The median period of follow-up was of 43 months (3–156 months). One patient presented with a recurrent abscess of the breast.

**Conclusion:**

Our study found that clinical examination and radiological imaging were not specific. Positive cultures for Koch bacillus or histological confirmation are mandatory for the diagnosis. A meta-analysis of the existing cases is needed.

## Introduction

Despite the advances in treatment, Tuberculosis (TB) is still a global health problem, responsible for 1.3 million deaths in 2012 [1]. All the organs can be affected by TB, with some disparity in the incidence in favor of the Lung. The diagnoses of the extrapulmonary locations, especially in their primary form, can be challenging. Breast TB (BTB) is an uncommon location first described in 1829 [2]. The reported incidence is less than 1% of all breast disease [3, 4].

Due to the disease's rarity, the available data is scarce, making the diagnosis and management of affected patients challenging. Many questions remain unanswered. Are there any specific clinical or radiological signs? What is the ideal diagnostic modality? Is there a place for surgery in the era of antitubercular treatment?

Our aim through this case series is to report our experience and discuss diagnoses and management modalities.

## Case presentation

We conducted a retrospective case series of twelve Caucasian North African patients with breast tuberculosis seen in our institution for over 15 years, from 2004 to 2019.

The mean age of our patients was 42 years old (22–63 years old). All the patients included were female. Among these women, one was breastfeeding at the moment of diagnosis (8%). Constitutional symptoms of tuberculosis, such as fever, night sweats, and weight loss, were reported by one patient (8%). No immunosuppressive conditions were present (Table 1).

**Table 1:** Baseline characteristics of patients (*n*=12)

Patient characteristics	*N* (%)
Age (mean, y)	42 (22–63)
Martial status	
Single*	2 (17)
Married*	10 (83)
Employment status	
Unemployed*	12 (100)
Employed*	0 (0)
Menopausal status	
Premenopausal*	8 (67)
Postmenopausal*	4 (33)
Location	
Rural*	4 (33)
Urban*	8 (67)
Pregnancy at time dignosis	0 (0)
Active breastfeeding	1 (8)
Multiparity	5 (42)
Coexistent pulmonary tuberculosis	1 (8)
Contact history of tuberculosis	0 (0)
BCG vaccination	12 (100)

The classic presentation was a breast lump found in half of the cases (50%). Four patients had tender nodules (33%), one patient had a breast condensation (8%), and a breast abscess in one case (8%). We noticed a serous nipple discharge in one case (8%). In seven cases, the examination of the axilla found suspicious lymphadenopathy (58%). We reported three cases of skin retraction (25%) and 1 case of edema and skin erythema (8%). One woman had a fistula (8%). Bilateral involvement was present in one case (8%). Multifocal tumors in the upper outer quadrant of the right breast were present in one patient (8%). The lesions were in the right breast in eight cases (66%) and the left one in three cases (25%). Eight of the tumors were in the upper outer quadrant of the breast (57%), and two extended to the outer quadrant (14%). The lump was in the retro-areolar region in two patients (14%). The lesions involved the internal quadrant in two patients (14%), one in the lower part (7%), and the other in the upper (7%). The average size of the tumors was 39 mm (15-80 mm). Clinically, lesions suspicious of breast carcinoma were present in seven patients (58%). Nine patients had a mammogram (58%).

The imaging findings varied; we found five speculated ill-defined mass (41%) with an average size of 33 mm (25–60 mm). Two cases of well-limited masses were described (17%) and two other patients presented with an asymmetry of density (17%).

**Fig. 1:**
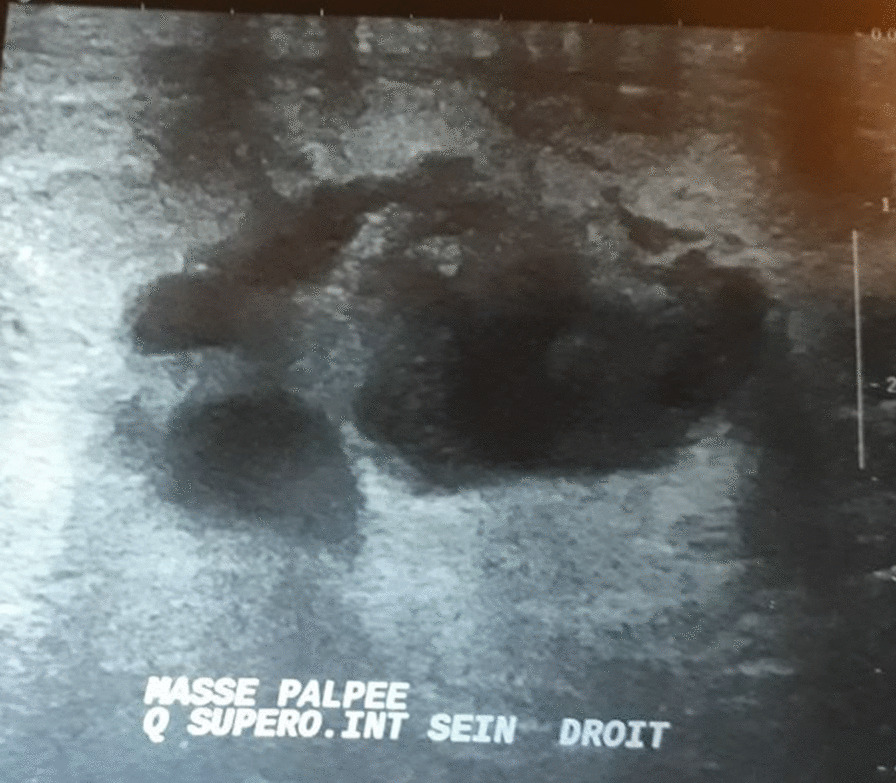
Suspicious multiloculated cyst with intracavitary vegetation

**Fig. 2:**
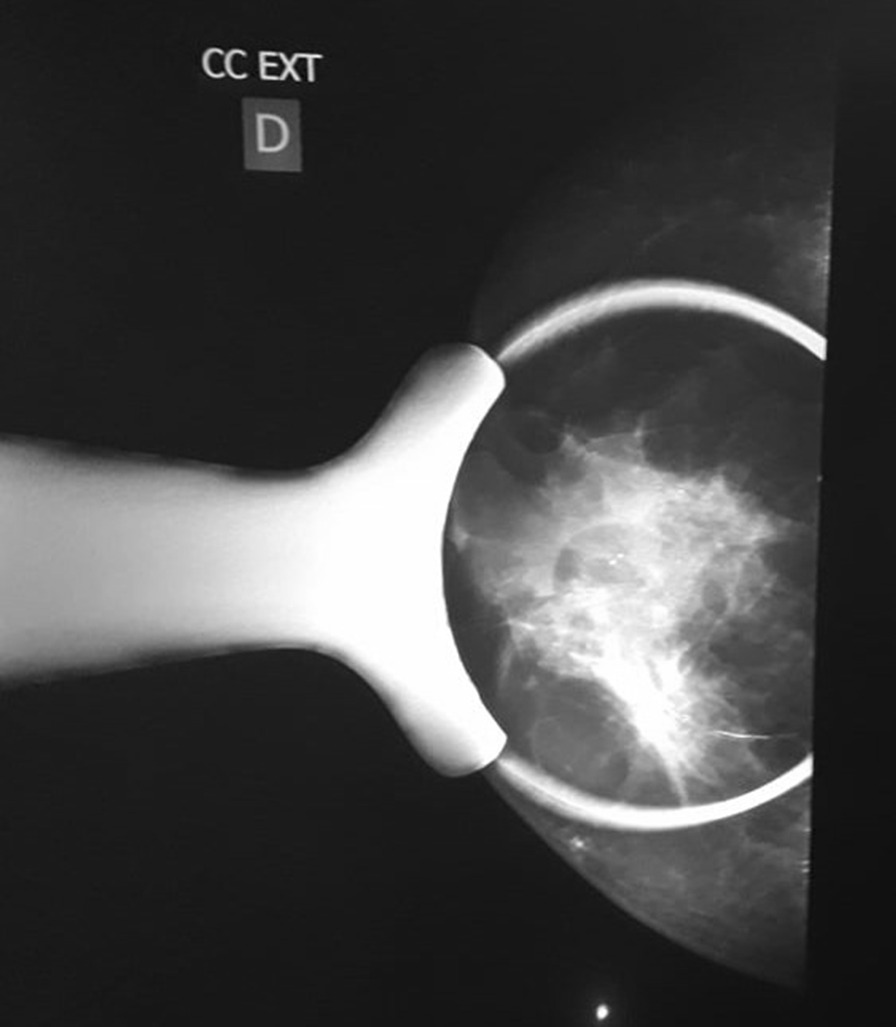
Speculated retro-areolar opacity

Six cases underwent an ultrasonography of the breast. Its findings revealed a suspicious ill-circumscribed nodule in four patients (33%) with an average size of 37.5 mm (10–60 mm). In one case, a hypo echoic range was present (8%), and in the other one, we reported two well-limited nodules in the same breast mimicking a fibro adenoma (8%). (Figs. 1, 2)

All patients had a chest X-ray, showing lesions associated with pulmonary tuberculosis in one patient (8%) (Table 2).

**Table 2:** Radiological findings

Radiological findings	*N* (%)
Chest X-ray	12 (100)
Normal*	11 (92)
Pleural effusion*	1 (8)
Ultrasonographic findings	6 (50)
Ill-circumscribed nodule*	4 (34)
Well-limited nodule*	1 (8)
Hypoechoic mass*	1 (8)
Mammographic findings	7 (58)
Spiculated mass*	5 (41)
Circumscribed mass*	2 (17)
Asymmetric density*	2 (17)

Two patients (17%) underwent a fine needle aspiration cytology that was non-conclusive. Four patients had a core biopsy. The frozen section results only ruled out malignancy without confirming BTB (33%).

Five patients had lumpectomy (42%), and seven women underwent drainage of the abscess and the biopsy of its hull (58%).

**Fig. 3:**
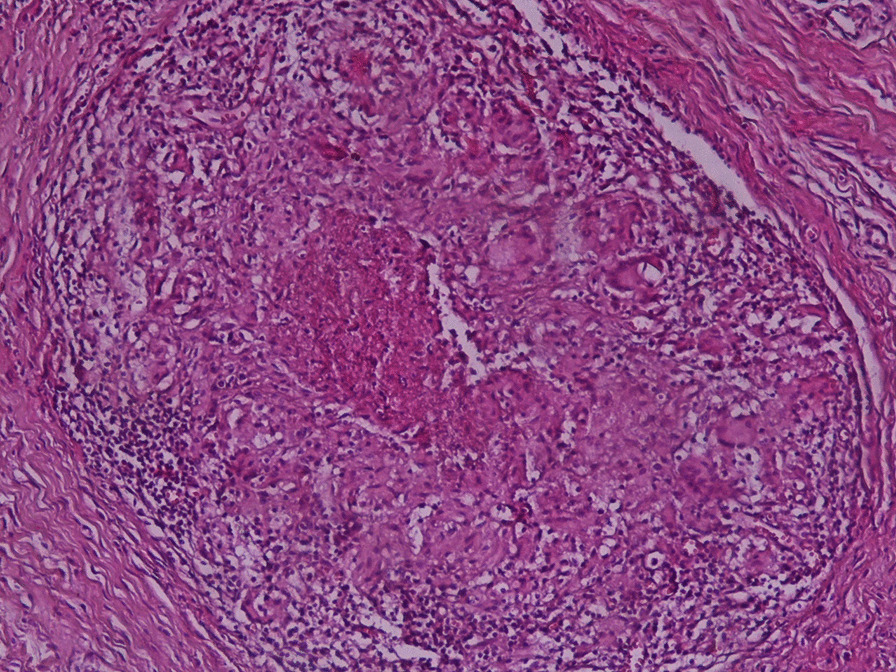
histologic picture of granulomatosis and caseous necrosis

The final histological diagnosis of tuberculosis was confirmed by an association of epithelioid cell granuloma and caseous necrosis (Fig. 3).

All the patients were referred to the infectious diseases department to undergo an appropriate treatment and follow up. All of them undertook antitubercular therapy. The median period of follow-up was of 43 months (3–156 months). Eleven patients had an uncomplicated postoperative course (92%). One patient presented with a recurrent abscess and was treated with drainage and a 9-month antitubercular protocol (8%).

## Discussion

BTB usually affects young women of reproductive age with a mean age ranging from 32,2 and 40 years old [4, 5]. It can also affect children, older women, and men [5]. The disease is also more common among breastfeeding, multiparous women, and that can be explained by the fact that the breast is more sensitive to infection due to the increase of vascularity and repeated trauma [3]. In our study, the mean age was 42 years old. Five of our patients were multiparous, and one was breastfeeding.

Constitutional signs of tuberculosis, such as fever, weight loss, night sweats, are present in less than 20% of the literature cases [6]. As in most cases, the patient reports a chief complaint related to the breast; physicians rarely looked after those symptoms. In our study, only one patient reported high fever and night sweats.

BTB is usually a unilateral disease with no difference between both sides [5, 7, 8]. Some authors reported a predominance of a side over another [5, 9]. In our study, 66% of the patients had unilateral disease affecting the right breast.

The most common clinical presentation reported was in the form of a lump [10]. This lump was usually ill-defined and irregular [10] and is located in the central and upper quadrant [6]. In this form, BTB can easily mimic breast carcinoma [6, 11]. Other signs have been reported, such as fistula formation, nipple or skin retraction, and breast discharge [4]. The lump may be associated with breast swelling and abscess formation, skin ulceration, and diffuse mastitis [4, 6]. Recurrent abscess of the breast that did not respond to surgical drainage and usual antibiotics should raise suspicion [6].

Our results are concordant with the literature.

### Is radiology enough for diagnosis?

The most commonly used radiological tool was breast ultrasound (US). Mammography was used in most of the studies for women over 35 years old [2, 4]. BTB in imaging can present itself in almost all aspects, but it was mainly described as lump/nodules [3]. Other common presentations are fluid collections, enlarged axillary lymph nodes, and inflammatory signs [4, 5, 10].

However, imaging does not help distinguish between BTB and the other differential diagnoses such as carcinoma, granulomatosis, or breast infections [12] due to the lack of specificity. Radiological workup helped determine the spread of the disease [12].

### What is the ideal diagnostic method? (Fig. 4)

**Fig. 4:**
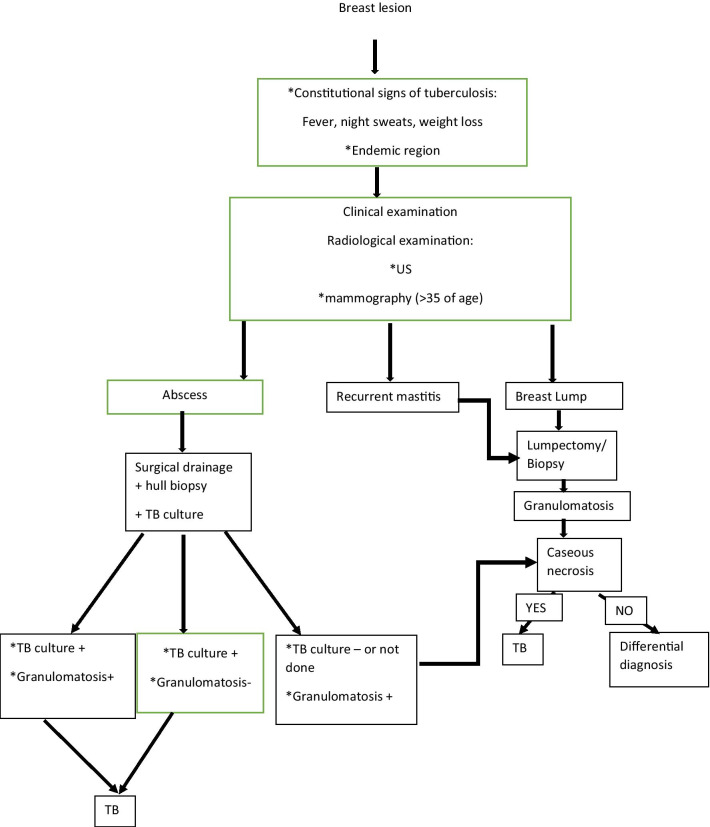
Algorithm for the diagnosis of breast tuberculosis

The diagnosis of BTB by isolating Mycobacterium Tuberculosis (MT) in bacteriological culture or using Ziehl Neelson stain is the gold standard [2, 13]. However, in BTB, MT is found in one-fifth of the cases. In less than 12% of the patients, acid-fast bacteria were identified [12]. That is why; some authors agreed that the histological finding of granulomatosis associated with caseous necrosis is sufficient for the diagnoses [3, 14–16]. Fine needle aspiration (FNA) is the most used diagnostic method with a 73% specificity when it associates granulomatosis and caseous necrosis, according to Baharoon et al. [12]. In some other studies, FNA was very often inconclusive; sensitivity for FNA was 28% instead of 94% for histology. Acid-fast bacteria were present in 10.3% of the FNA specimens and 29.7% of the histology specimens [17]. Longman et al. [2], found that FNA was able to diagnose BTB in 18 % of the cases (6/33 patients). In our study, two patients had FNA that were inconclusive.

When facing an inconclusive FNA, obtaining a larger histologic sample may be advocated, especially when the need to rule out other breast diseases such as carcinoma is on the line [2]. In our series, all the patients had a surgical biopsy that showed granulomatosis associated with caseous necrosis.

### Is all breast granulomatosis BTB?

Another challenging differential diagnosis is the other granulomatous conditions. Longman et al. [2] described a schematic diagram for the differentiation and diagnosis of the breast's granulomatous disease. They first suggested an immediate referral to the TB clinic. To rule out Wegener's granulomatosis, they recommended looking for antineutrophilic cytoplasm antibodies directed against MPO and PR3.

A history of sarcoidosis, a positive serum ACE, or appropriate chest X-ray findings can raise breast sarcoidosis suspicions. The diagnosis of idiopathic granulomatosis is possible after ruling out the three previous breast granulomatosis diseases [2]. In the last differential diagnosis, the primary treatment is steroids, which can flare up tuberculosis; that is why an antitubercular treatment can be warranted [12].

### Is there a place for surgery?

The treatment of BTB relies on courses of antitubercular antibiotics in a specialized unit. Surgery is mainly a diagnostic tool allowing adequate tissue sampling [11]. Women with a locally advanced or resistant disease could benefit from a segmental mastectomy can also be offered [18]. Total mastectomy can also be provided for women with locally advanced disease with painful large ulcerative lesions [19].

In our study, all the patients had surgery for diagnosis purposes. None had to undergo mastectomy for resistant or locally advanced disease.

Our study has shown some limitations, the retrospective nature of the research and the low number of patients. However, we do attach weight to its clinical implications in the view of the scarcity of the disease and the paucity of literature.

### Conclusion

Our findings were similar to the literature, showing the low specificity of clinical and radiological findings. Physicians in endemic countries should be aware of this differential diagnosis when facing breast abscess or other breast conditions, especially in front of the patient with recurrent abscess, constitutional symptoms of tuberculosis, history, or active tuberculosis. The final diagnosis relies on BK's positive culture or the association of epithelioid cell granuloma and caseous necrosis in the histology examination.

The treatment depends on antitubercular antibiotics. Surgery is an excellent therapeutic tool in case of recurrent abscess or resistant disease.

A meta-analysis of the existing cases can help determine more specific clinical or radiological signs and give a higher grade of recommendation for managing the disease.

## Data Availability

Approval from the medical committee of our institution was obtained beforehand. Baseline characteristics' included Age, Gender, gestational status, and past medical history. The clinical data studied were chief complaints, breast examination, and lump size. The radiological data were assessed from Ultrasound and mammography reports when made. The tissue sampling methods used were Fine needle biopsy, core needle biopsy, or surgical excision/biopsy. The diagnoses were confirmed by the combination of clinical suspicion, histology findings, and clinical response to treatment. As our institution is an anticancer center, we do not commonly observe tuberculosis and perform diagnostic tests for tuberculosis only in selected cases. All the diagnosed patients were referred to an infectious disease department. All of them were included in the screening program for breast cancer, allowing us to follow up further. The literature review was made using Pubmed and ScienceDirect. Most of the studies reporting breast tuberculosis in English from 2010 were reviewed. Excel 2016 version was used for the statistical analysis. Descriptive analysis was used to assess the demographic findings. The results are presented as mean ±SD for continuous variables and number/percentage for categorical variables.
